# Spanish Translation and Validation of the COVID Stress Scales in Peru

**DOI:** 10.3389/fpsyg.2022.840302

**Published:** 2022-03-28

**Authors:** Martin Noe-Grijalva, Anali Polo-Ambrocio, Karla Gómez-Bedia, Tomás Caycho-Rodríguez

**Affiliations:** ^1^Faculty of Health Sciences, Universidad César Vallejo, Trujillo, Peru; ^2^Faculty of Health Sciences, Universidad Privada del Norte, Lima, Peru

**Keywords:** COVID-19, stress, reliability, invariance, validity

## Abstract

The objective of the study was to translate and validate the COVID Stress Scales (CSS-36) into Spanish in Peru. Around 1,424 people, selected through a non-probabilistic sampling, participated in the study. Factor analysis confirmed an initial six-dimensional factorial structure of the CSS-36. Reliability by internal consistency was good for the dimensions of fear of danger, socioeconomic consequences, xenophobia, fear of contamination, traumatic stress, and compulsive control. In addition, the factorial structure of scale has been shown be strictly invariant for both males and females. The Spanish version of the CSS-36 has evidence of validity, reliability, and invariance to measure COVID-19 stress in a Peruvian sample.

## Introduction

SARS-COV2 is the cause of a potentially fatal disease, called coronavirus disease (COVID-19), which constitutes a major public health problem in the world (Gallegos et al., 2020; Rothan and Byrareddy, 2020), with 176,353,405 confirmed cases and 3,814,010 deaths globally as of June 15, 2021 (Coronavirus Resource Center, 2021). In December 2019, the first cases of pneumonia of unknown origin were identified in Wuhan, China. On January 12, the WHO confirmed a new coronavirus was the cause of pneumonia in Wuhan (Fitzgerald and Wong, 2020). By the end of February 2020, the SARS-COV2 virus had already spread rapidly within China and 28 other countries. On January 13, the first case outside of China was reported, occurring in Thailand, and on January 19, South Korea reported its first documented case of COVID-19. In Europe, the first three cases were detected in France on January 24, 2020; while the first death was reported on February 15 in that same country (Stoecklin et al., 2020). As of February 21, 47 cases of COVID-19 had already been confirmed in the European Region of WHO (Spiteri et al., 2020). In Latin America and the Caribbean, the first case of COVID-19 was reported on February 25, 2020, in Brazil (Rodriguez-Morales et al., 2020a) and then its presence was reported in different countries throughout the region (Rodríguez-Morales et al., 2020b). In Peru, there were a total of 200,4252 cases and 188,921 deaths, reported as of June 15, 2021, where the departments of Arequipa, La Libertad, and Piura have the highest percentage of reported cases (Ministerio de Salud, 2021).

This scenario has been directly affecting different aspects of our daily lives, increasing levels of stress, depression, and anxiety. This seems to be associated with coping strategies adopted, level of awareness about the disease, sociodemographic variables (such as gender and educational level), people’s habits, household characteristics, the way in which people use media of information, uncertainty regarding the disease, temporary restrictions on our freedom of movement and relationship with respect to our family and friends (Scholten et al., 2020; Passavanti et al., 2021). Along the same lines, it has also been suggested that a greater number of hours is related to a lower fear of contagious diseases and that national measures to mitigate the pandemic moderated a negative relationship between resilience and anxiety (Moret-Tatay and Murphy, 2022). Prevalence of mental problems worldwide the before COVID-19 pandemic varied between countries, with one study finding 28% of the population had depressive symptoms, 26.9% anxiety symptoms, 24.1% post-traumatic stress symptoms, 36.5% stress symptoms, 50% psychological distress, and 27.6% sleep problems (Nochaiwong et al., 2021). In addition, problems of alcohol and drug abuse, grief reactions, aggravation of previous mental disorders, and post-traumatic stress disorder have been reported (Hossain et al., 2020; Vindegaard and Benros, 2020). This situation can also be seen in Peru, where between 30 and 40% of people have presented symptoms of depression, anxiety, and post-traumatic stress during the pandemic (Palomino-Oré and Huarcaya-Victoria, 2020). The presence of symptoms of anxiety and depression is related to concern for their loved ones (Vásquez et al., 2020), which means this pandemic may affect more people than indicated in the number of confirmed cases (Prieto-Molinari et al., 2020).

Regarding COVID-19 stress, review and meta-analysis studies have reported a prevalence between 29.6% (95% CI 24.3–35.4) to 43% (95% CI 37–49) in the general population (Salari et al., 2020; Al Maqbali et al., 2021). COVID-19 stress syndrome is characterized by a network of interconnected symptoms, such as fear of the dangerousness of COVID-19 in one’s family, socioeconomic concerns, xenophobia, symptoms of traumatic stress, and compulsive control, as well as seeking comfort (Taylor et al., 2020a). Different studies have suggested stress related to COVID-19 has been linked to increased fear of COVID-19, intolerance to uncertainty, depression, anxiety, neurasthenia, and hypochondria (Bakioğlu et al., 2020; Yan et al., 2021). In addition, COVID-19 stress significantly predicts optimism-pessimism, psychological inflexibility, and psychological problems (Arslan et al., 2020). It is also related to panic buying, excessive avoidance, and higher levels of distress and lowered adaptive coping during isolation (Taylor et al., 2020a). People with symptoms of COVID-19 stress tend to avoid public places, where they can be infected or encounter the disease and are more afraid of (and even avoid) avoid people who might be infected with COVID-19, such as health care workers (Taylor et al., 2020c,d). Similarly, people with greater COVID-19 stress are more likely to engage in self-destructive coping behaviors, such as overeating, drug and alcohol abuse, and over-shopping online (Taylor et al., 2020e, 2021). To a lesser extent, COVID stress was linked to belief in COVID-19 conspiracy theories and anti-vaccination attitudes related to COVID-19 (Taylor, 2021).

For an adequate measurement of COVID-19 stress, the COVID Stress Scales (CSS-36; Taylor et al., 2020e) were recently developed, which evaluate stress related to COVID-19 based on the definition presented by Taylor et al. (2020e) which has five factors: fears of danger and contamination, fears of socioeconomic consequences, xenophobia, compulsive checking including seeking comfort, and symptoms of traumatic stress. Taylor et al. (2020e) reported adequate psychometric results for the CSS-36, further suggesting that it can easily adapt to future pandemics. This study was carried out with representative samples from Canada (*N* = 3,479) and the United States (*N* = 3,375). Another study sought to validate the Persian version of the CSS-36 in a clinical sample, reporting adequate psychometric properties (Khosravani et al., 2021). Another study adapted and validated an Arabic version of the CSS-36 in Egyptian and Saudi university students (*N* = 1,080), and also reported satisfactory properties of validity and reliability (Abbady et al., 2021). However, there is no adaptation and validation of the CSS-36 into Spanish.

Psychometric studies of the Persian and Arabic versions of the CSS-36 are based on Classical Theory of Tests (CTT), which emphasizes the evaluation of internal consistency and construct validity of an instrument in a general way (Hunsley and Mash, 2008); however, CTT does not specify the relationship between latent ability supposedly measured and result observed in the test (Leenen, 2014). On the other hand, Item Response Theory (IRT), can evaluate a functional relationship between values of the variable that the test measures and the characteristic curve of each item, which leads to having more precise scores determine a more accurate clinical diagnosis (Muñiz, 2010). However, previous psychometric studies have not included IRT, which could provide more information about COVID-19 stress. They have also not reported evidence of measurement invariance (MI). Evaluating MI is important in health sciences because it provides evidence that different groups attribute the same meaning to items on a scale (Caycho, 2017). This would allow people with a similar level in a psychological trait to provide similar responses to the scale, regardless of the group to which they belong (Milfont and Fischer, 2010). Thus, MI is a prerequisite for making comparisons between different groups (Putnick and Bornstein, 2016). Absence of MI would not allow us to be certain that a construct has the same meaning in different groups and, therefore, the conclusions drawn between groups can be erroneous and biased. This would not reflect true differences in how individuals from different groups respond to items on a given scale (Byrne, 2008; Dimitrov, 2010).

For the reasons given above, this study aimed to adapt and evaluate the psychometric evidence of a Spanish version of the CSS-36 in the Peruvian population. The adaptation was done using Latin American Spanish, particularly as spoken in Peru. The CSS-36 was adapted into this particular regional Spanish in order for sociolinguistic variation to be taken into account during the adaptation process to ensure its interpretations would be faithful to the original (Peterson et al., 2017). In this sense, although it is valuable to obtain pan-dialectal versions, additional linguistic adaptations are necessary for certain cultural contexts (Squires et al., 2013). As for the statistical analysis, we evaluated evidence of validity based on internal structure, reliability by internal consistency, invariance of the measurement by sex and the characteristics of difficulty and discrimination of the items based on IRT. Based on previous evidence, a five-factor structure is expected to present an adequate fit and show evidence of reliability (Taylor et al., 2020e; Abbady et al., 2021; Khosravani et al., 2021). Similarly, it would be expected that, based on IRT, a greater presence of the latent trait, namely COVID-19 stress, will be required to respond to the higher response categories, as occurs in other instruments used during the COVID-19 pandemic (Caycho-Rodríguez et al., 2021c,d,e). While there is no prior evidence of MI for the CSS-36, it would be expected to be invariant between different sex groups, as has happened with other scales which measure mental health indicators during the current pandemic (e.g., Caycho-Rodríguez et al., 2021b). Previous literature indicates that women had a higher prevalence of stress symptoms during the COVID-19 pandemic than men (Pieh et al., 2020; Xiong et al., 2020; Kolakowsky-Hayner et al., 2021). Having a sex-invariant measure will allow us to assess the disparities observed in COVID-19 vulnerability between men and women and to better understand the impact of sex on incidence of stress, as well as adapt treatment (Gebhard et al., 2020). Findings of this study will contribute to the body of valid and reliable information about the impact of the COVID-19 pandemic on some mental health symptoms in the Peruvian population. In addition, the online nature and results of the study provide an opportunity to include the CSS-36 in an online self-assessment system that motivates people to use mental health services if they see the need (Lee et al., 2021). In this sense, online assessments enable immediate notification about mental health status, which can improve users’ mental health literacy and encourage them to seek help (Van Agteren et al., 2020).

## Materials and Methods

### Participants

Present study was carried out in six cities in Peru (Lima, Trujillo, Chimbote, Huaraz, and Chiclayo). Around 1,424 people participated, with ages ranging from 18 to 42 years old (*M* = 28.7 years old, *SD* = 12.36), and they were selected through a non-probabilistic sampling. About 56.2% were female (*N* = 800) and 43.8% male (*N* = 624), most were single (73.7%), 29% reside in Huaraz, 40.7% had no job, and 36.2% have completed university studies. In addition, 20.1% had been diagnosed with COVID-19 and 69.9% stated they had family or friends with COVID-19. Table 1 presents detailed information on the characteristics of the participants.

**Table 1:** Description of the characteristics of the participants.

Sex	Frequency	Percentage
Men	624	43,8
Women	800	56,2
*Marital status*		
Married	180	12,6
Cohabitant	156	11,0
Divorced	34	2,4
Single	1,049	73,7
Widowed	5	0,4
*Place of residence*		
Chimbote	348	24,4
Huaraz	413	29,0
Lima	399	28,0
Piura	39	2,7
Tarapoto	3	0,2
Trujillo	70	4,9
Others	152	10,7
*Employment status*		
Are out of work	580	40,7
Have a permanent or dependent job	463	32,5
Have a temporary job	381	26,8
*Educational level*		
Complete primary	9	0,6
Incomplete secondary	33	2,3
Complete secondary	169	11,9
Incomplete technical studies	67	4,7
Complete technical studies	180	12,6
Incomplete university	451	31,7
Complete university	515	36,2
*Diagnosed with COVID-19*		
No	1,138	79,9
Yes	286	20,1
*Family or friend with COVID-19*		
No	429	30,1
Yes	995	69,9

### Instruments

#### Sociodemographic Questionnaire

An *ad hoc* questionnaire was used which included questions about sex, age, marital status, place of residence, employment status, and educational level, whether they have been diagnosed with COVID-19, and if participant has had any family or close friends diagnosed with COVID-19.

#### COVID Stress Scales

The CSS-36 was developed to assess COVID-19-related stress symptoms. It consists of 36 items; from item 1 to 24 each item has five answer options (0 = by no means to 4 = extremely) and from item 25 to 36 it has five answer options (0 = never to 4 Almost always). These items are related to symptoms presented in the last 7 days and grouped into five factors: (1) fears of danger and contamination, (2) fears about socioeconomic consequences, (3) xenophobia, (4) compulsive checking and search for reassurance, and (5) symptoms of traumatic stress related to COVID-19 (Taylor et al., 2020e).

### Procedure

First, the CSS-36 was translated into Spanish as spoken in Peru in according to recommendations for cross-cultural adaptation of self-report measures (Beaton et al., 2000):

1. Two bilingual native Spanish speakers (first was a mental health professional with knowledge of the subject and second was a professional translator with no knowledge of the subject) performed two independent translations from English to Spanish. From comparison of the two translations, an initial Spanish version of the CSS-36 was developed.
2. Initial Spanish version was translated into English by two freelance translators whose native language was English, but who spoke Spanish fluently.
3. The four translators mentioned above, together with two experts in the field and members of the research team, evaluated all translated versions and the original version, from which a preliminary version of the CSS-36 in Spanish was developed.
4. Preliminary version of the CSS-36 was administered to 10 adults to assess their comprehensibility and readability. Experts together with translators reviewed results of initial version to modify the CSS-36 items if necessary. Respondents did not suggest any modifications, which allowed us to have a final version of the CSS-36 in Spanish.

Table 2 shows a sample of items from the original English version and final Spanish translated version of the CSS-36.

**Table 2:** Sample items of the original English version and the final Spanish translated version of the COVID Stress Scales (CSS-36).

Factor	Items	Original version	Version translated into Spanish
Danger	d1	I am worried about catching the virus	Estoy preocupado (a) por contraer el virus.
Socio-economic consequences	s1	I am worried about grocery stores running out of food	Estoy preocupado (a) por que las tiendas de abarrotes se queden sin alimentos.
Xenophobia	x1	I am worried that foreigners are spreading the virus in my country	Me preocupa que los extranjeros estén propagando el virus en mi país.
Contamination	c1	I am worried that people around me will infect me with the virus	Me preocupa que las personas que me rodean me infecten con el virus.
Traumatic stress	t1	I had trouble sleeping because I worried about the virus	Tuve problemas para dormir porque me preocupaba el virus.
Checking	ch1	Social media posts concerning COVID-19	Publicaciones en redes sociales sobre COVID-19.

The final version of the CSS-36 was applied during the months of January and February 2021, a period in which greater restrictions established by the Peruvian government to try to mitigate impact of COVID-19 were announced. As part of these restrictions, all regions of Peru were categorized as high, very high, or extreme risk. All gatherings including family, social, cultural, and political events were banned. Citizens were also advised to follow all lockdown measures. During the period of application, a contagion rate of approximately 1,790 daily cases and a total rate of 8,855 deaths were observed (Ministerio de Salud, 2021).

An online form was created consisting of the sociodemographic questionnaire and the 36 items of the CSS-36 through the Google Forms platform, which was shared through social networks (Facebook and Instagram) and WhatsApp. The study was reviewed by both the Universidad César Vallejo and the Universidasd Privada del Norte. Approval was received from the Ethics Committee of the Universidad Privada del Norte in Peru (registration number: 20213002 dated January 10, 2021). As part of the consideration for the corresponding aspects of ethics and confidentiality, each participant was not asked for identification data that could expose them or violate their right to privacy. Therefore, the study does not involve any risk to participants.

### Data Analysis

For Confirmatory Factor Analysis (CFA) the *Diagonally Weighted Least Squares with Mean and Variance corrected* (WLSMV) estimator was used since the items are at the ordinal level (Brown, 2015). The evaluation of model fit was based on the chi-square test (*χ*^2^), root mean square error of approximation (RMSEA) index, and standardized root mean square residual (SRMR) index, whose values less than 0.05 indicate good fit, and between 0.05 and 0.08 are considered acceptable (Kline, 2015). In addition, the comparative fit index (CFI) and Tucker-Lewis index (TLI) were used, where values greater than 0.95 indicate good fit and greater than 0.90 an acceptable fit (Schumacker and Lomax, 2015). To evaluate internal consistency of the scale, *Cronbach’s alpha* coefficient (Cronbach, 1951), and omega coefficient (McDonald, 1999) were used (Viladrich et al., 2017).

Evaluation of invariance of the scale according to sex of participants was carried out in a sequential model of hierarchical variance. First the configural invariance (reference model) was evaluated, followed by metric invariance (equality of factorial loads), scalar invariance (equality of factorial and intercept loads), and finally strict invariance (equality of factorial loads, intercepts, and residues) was tested. To compare the sequence of models, firstly, the chi-square difference (Δ*χ*^2^) was used, where non-significant values (*p* > 0.05) suggest invariance between the groups. Secondly, a modeling strategy based on the differences in the RMSEA (ΔRMSEA) was used, where differences smaller than <0.015 show the invariance of the model between the groups (Chen, 2007). Use of RMSEA was included because it is much more robust to sample size and complexity of the model (Schermelleh-Engel et al., 2003) and works best in factorial models with ordinal data (Xia and Yang, 2019).

Item Response Theory -based analyses were performed with a Graduated Response Model (GRM; Samejima, 1997) specifically an extension of the 2-parameter logistic model (2-PLM) for ordered polytomous items (Hambleton et al., 2010). For model fit, the C2 test developed for ordinal items (Cai and Monroe, 2014) was used. The following adjustment criteria were used: RMSEA ≤ 0.05 (Maydeu-Olivares and Joe, 2014) and SRMSR ≤ 0.05 (Maydeu-Olivares, 2013). CFI and TLI values were considered using the same adjustment criteria (≥0.95) used in SEM models (Lubbe and Schuster, 2019).

For each item, two types of parameters were estimated: discrimination (*a*) and difficulty (*b*). Parameter *a* determines the slope on which responses to items change depending on level of the latent trait and *b* parameters determine how much of the latent trait the item requires to be answered with a given response. Since the CSS-36 has five response categories, there are four difficulty estimates, one per threshold. Estimates for these four thresholds indicate the level of latent variable at which an individual has a 50% chance of obtaining a score equal to or greater than a response category in particular. Information curves for items (IIC) and the test information curve (TIC) were also calculated.

Statistical analyses were performed using the “lavaan” package (Rosseel, 2012) for the CFA, the “semTools” package (Jorgensen et al., 2018) for factorial invariance and the “mirt” package for the GRM (Chalmers, 2012). In all cases, the RStudio environment (RStudio Team, 2018) was used for R (R Core Team, 2019).

## Results

### Descriptive Analysis of Items

In Table 3, item 2 [“I am worried that I cannot keep my family safe from the virus. (Me preocupa no poder mantener a mi familia a salvo del virus)”] has the highest average score in this sample (*M* = 2.52). In contrast, item 30 [“I had bad dreams about the virus. (Tuve pesadillas sobre el virus)”] has the lowest average score in the same group of participants (*M* = 0.69).

**Table 3:** Descriptive analysis of items.

Items	*M*	*SD*	*g1*	*g2*
1. I am worried about catching the virus [Estoy preocupado (a) por contraer el virus.]	1.87	1.02	0.09	−0.33
5. I am worried that basic hygiene (e.g., handwashing) is not enough to keep me safe from the virus [Me preocupa que la higiene básica (por ejemplo, lavarme las manos) no sea suficiente para mantenerme a salvo del virus.]	1.97	1.02	0.07	−0.43
4. I am worried that our healthcare system is unable to keep me safe from the virus [Me preocupa que nuestro sistema de salud no pueda mantenerme a salvo del virus.]	2.49	1.06	−0.41	−0.41
2. I am worried that I cannot keep my family safe from the virus [Me preocupa no poder mantener a mi familia a salvo del virus.]	2.52	1.01	−0.31	−0.39
3. I am worried that our healthcare system will not be able to protect my loved ones [Me preocupa que nuestro sistema de salud no pueda proteger a mis seres queridos.]	2.71	1.04	−0.58	−0.22
6. I am worried that social distancing is not enough to keep me safe from the virus [Me preocupa que el distanciamiento social no sea suficiente para mantenerme a salvo del virus]	2.28	1.05	−0.12	−0.53
7. I am worried about grocery stores running out of food [Estoy preocupado (a) por que las tiendas de abarrotes se queden sin alimentos.]	1.63	1.15	0.21	−0.75
10. I am worried about grocery stores running out of cold or flu remedies [Me preocupa que el remedio para el resfriado y la gripe se termine en los supermercados o centros autorizados o centros de abastos.]	1.67	1.17	0.15	−0.79
12. I am worried about pharmacies running out of prescription medicines [Me preocupa que los medicamentos con receta médica se agoten en las farmacias.]	1.86	1.14	0.06	−0.78
11. I am worried about grocery stores running out of water [Me preocupa que en los almacenes de abarrotes y/o supermecados se acabe el agua.]	1.61	1.23	0.22	−0.94
9. I am worried about grocery stores running out of cleaning or disinfectant supplies [Me preocupa que los supermercados se queden sin productos de limpieza o desinfectantes.]	1.58	1.15	0.19	−0.81
8. I am worried that grocery stores will close down [Estoy preocupado (a) de que las tiendas de comestibles lleguen a cerrar sus puertas.]	1.69	1.15	0.07	−0.88
13. I am worried that foreigners are spreading the virus in my country [Me preocupa que los extranjeros estén propagando el virus en mi país.]	2.02	1.24	−0.04	.−95
16. If I met a person from a foreign country, I would be worried that they might have the virus [Si conociera a una persona de un país extranjero, me preocuparía que pudiera tener el virus.]	1.89	1.13	0.06	−0.69
15. I am worried about coming into contact with foreigners because they might have the virus [Me preocupa entrar en contacto con extranjeros porque pueden tener el virus.]	1.83	1.17	0.09	−0.79
18. I am worried that foreigners are spreading the virus because they are not as clean as we are [Me preocupa que los extranjeros propaguen el virus porque no son tan aseados como nosotros.]	1.61	1.23	0.27	−0.91
14. If I went to a restaurant that specialized in foreign foods, I would be worried about catching the virus [Si fuera a un restaurante especializado en comida extranjera, me preocuparía contraer el virus.]	1.51	1.19	0.33	−0.82
17. If I was in an elevator with a group of foreigners, I’d be worried that they are infected with the virus [Si estuviera en un ascensor con un grupo de extranjeros, me preocuparía que estén infectados con el virus.]	1.91	1.18	0.07	−0.83
21. I am worried that people around me will infect me with the virus [Me preocupa que las personas que me rodean me infecten con el virus.]	1.94	1.08	0.10	−0.65
19. I am worried that if I touched something in a public space (e.g., handrail, door handle), I would catch the virus [Me preocupa que si toco algo en un espacio público (por ejemplo, pasamanos, manija de la puerta), contraiga el virus.]	1.95	1.08	0.14	−0.66
20. I am worried that if someone coughed or sneezed near me, I would catch the virus [Me preocupa que si alguien tosiera o estornudara a mi lado, contraería el virus.]	2.25	1.05	−0.07	−0.68
22. I am worried that I might catch the virus from handling money or using a debit machine [Me preocupa contraer el virus al manejar dinero o al usar una máquina de débito.]	1.93	1.07	0.13	−0.59
23. I am worried about taking change in cash transactions [Me preocupa recibir cambio en las transacciones en efectivo.]	1.81	1.07	0.15	−0.57
24. I am worried that my mail has been contaminated by mail handlers [Me preocupa que mi correo haya sido contaminado por manipuladores de correo.]	1.72	1.07	0.27	−0.54
27. I had trouble sleeping because I worried about the virus [Tuve problemas para dormir porque me preocupaba el virus.]	1.24	1.07	0.55	−0.35
30. I had bad dreams about the virus [Tuve pesadillas sobre el virus.]	0.69	0.96	1.33	1.05
28. I thought about the virus when I did not mean to [Pensé en el virus cuando no quise.]	1.19	1.02	0.59	−0.22
26. Disturbing mental images about the virus popped into my mind against my will [Aparecieron imágenes pertubardoras en mi mente sobre el virus sin desearlo.]	0.87	1.03	1.01	0.22
25. I had trouble concentrating because I kept thinking about the virus [Tuve problemas para concentrarme porque seguía pensando en el virus.]	0.93	1.02	0.87	0.06
29. Reminders of the virus caused me to have physical reactions, such as sweating or a pounding heart [Los recordatorios del virus me hicieron tener reacciones físicas, como sudoración o latidos cardiacos fuertes.]	0.88	1.05	1.04	0.32
36. Social media posts concerning COVID-19 [Publicaciones en redes sociales sobre COVID-19.]	1.75	1.10	0.16	−0.71
33. YouTube videos about COVID-19 [Vídeos de YouTube sobre COVID-19.]	1.39	1.06	0.45	−0.37
35. Seeking reassurance from friends or family about COVID-19 [Buscar tranquilidad de amigos o familiares sobre COVID-19.]	1.82	1.07	0.12	−0.53
34. Checking your own body for signs of infection (e.g., taking your temperature) [Revisar su propio cuerpo en busca de signos de infección (p. ej., tomar la temperatura).]	1.39	1.05	0.35	0.56
32. Asking health professionals (e.g., doctors or pharmacists) for advice about COVID-19 [Pedir consejo a los profesionales de la salud (por ejemplo, médicos o farmacéuticos) sobre COVID-19.]	1.56	1.05	0.27	−0.43
31. Searched the Internet for treatments for COVID-19 [Buscó tratamientos en internet para COVID-19.]	1.26	1.08	0.57	−0.36

*M* = mean; *SD* = standard deviation; *g1* = skewness; *g2* = kurtosis. The first 24 items use a scale from 0 [Not at all (De ningún modo)] – 4 [Extremely (Extremadamente)] and for the Spanish version the inner values were given the descriptions 1 (Ligeramente), 2 (Moderadamente), and 3 (Muy). The final 12 items use a scale from 0 [Never (Nunca)] – 4 [Almost always (Casi siempre)] and for the Spanish version the inner values were given the descriptions 1 (Raramente), 2 (Algunas veces), and 3 (Frecuentemente).

Regarding indices of asymmetry and kurtosis, it is observed all the items present adequate indices (As < ±2; Ku < ±7), according to the criteria of Finney and DiStefano (2006).

### Validity Based on Internal Structure and Reliability of the CSS-36

Table 4 shows the evaluation of the adjustment indices for two models: model 1 made up of six factors (Fears about danger of COVID-19, Fears about sources of contamination related to COVID-19, COVID-19 xenophobia, fears about the personal, social, and economic consequences of COVID-19, control related to COVID-19, and traumatic stress symptoms related to COVID-19) as initially postulated by Taylor et al. (2020e) based on a review of relevant literature and consultation with experts; model 2: final model consisting of five factors (Fears of danger and contamination, Fears about socioeconomic consequences, Xenophobia, Compulsive checking with search for reassurance, and symptoms of traumatic stress due to COVID-19) as proposed after psychometric analyses by Taylor et al. (2020e).

**Table 4:** Adjustment indices of both models.

Model	*χ* ^2^	df	*p*	CFI	TLI	SRMR	RMSEA [90%CI]
Model 1	4459.05	579	0.000	0.96	0.96	0.043	0.070 [0.068–0.071]
Model 2	7031.66	584	0.000	0.94	0.93	0.058	0.089 [0.087–0.091]

Model 1 = six items; Model 2 = five items; *χ*^2^, Chi square; df, degrees of freedom; SRMR, standardized root mean square residual; TLI, Tucker-Lewis index; CFI, comparative fit index; and RMSEA, root mean square error of approximation.

Model 1 with six related dimensions presents adequate adjustment indices in the total sample of participants [*χ*^2^ = 4459.05; df = 579; *p* = 0.000; RMSEA = 0.070 (90% CI 0.068–0.071); SRMR = 0.043; CFI = 0.96; and TLI = 0.96]. It can also be seen that all the items in model 1 have high factorial loads in the factors that correspond to them and the relationship between their dimensions is moderate (see Table 5). In contrast, model 2 with five related dimensions has worse adjustment indices [*χ*^2^ = 7031.66; df = 584; *p* = 0.000; RMSEA = 0.089 (90% CI 0.087–0.091); SRMR = 0.058; CFI = 0.94; and TLI = 0.93]. Therefore, for the following statistical analyses, model 1 with six related dimensions was used.

**Table 5:** Factor weights and reliability of the six-dimensional model 1.

Items	D	SE	X	C	T	CH
	λ (error)	λ (error)	λ (error)	λ (error)	λ (error)	λ (error)
E1	0.79 (0.38)					
E5	0.85 (0.28)					
E4	0.82 (0.33)					
E2	0.78 (0.39)					
E3	0.83 (0.31)					
E6	0.87 (0.25)					
E7		0.86 (0.26)				
E10		0.84 (0.29)				
E12		0.91 (0.17)				
E11		0.92 (0.15)				
E9		0.89 (0.21)				
E8		0.92 (0.16)				
E13			0.84 (0.29)			
E16			0.86 (0.26)			
E15			0.93 (0.14)			
E18			0.89 (0.21)			
E14			0.87 (0.25)			
E17			0.89 (0.21)			
E21				0.89 (0.22)		
E19				0.84 (0.29)		
E20				0.86 (0.26)		
E22				0.93 (0.14)		
E23				0.92 (0.15)		
E24				0.91 (0.17)		
E27					0.91 (0.17)	
E30					0.92 (0.16)	
E28					0.86 (0.26)	
E26					0.86 (0.26)	
E25					0.83 (0.32)	
E29					0.88 (0.23)	
E36						0.76 (0.42)
E33						0.77 (0.42)
E35						0.73 (0.47)
E34						0.82 (0.32)
E32						0.69 (0.52)
E31						0.70 (0.52)
*Correlations among the COVID Stress Scales*						
Danger (D)	-	0.61	0.62	0.73	0.44	0.55
Socio-economic consequences (SE)		-	0.71	0.69	0.45	0.52
Xenophobia (X)			-	0.81	0.44	0.53
Contamination (C)				-	0.53	0.61
Traumatic stress (T)					-	0.72
Compulsive checking (CH)						-
*Internal consistency*						
Alpha	0.92	0.96	0.95	0.96	0.95	0.88
Omega	0.89	0.95	0.95	0.96	0.94	0.87

In Table 5, the dimensions of Danger (*α* = 0.92; *ω* = 0.89), Socioeconomic consequences (*α* = 0.96; *ω* = 0.95), Xenophobia (*α* = 0.95; *ω* = 0.95), Contamination (*α* = 0.96; *ω* = 0.96), Traumatic stress (*α* = 0.95; *ω* = 0.94), and Compulsive control (*α* = 0.88; *ω* = 0.87) have adequate reliability indices.

### Factorial Invariance According to Sex

As shown in Table 6, the factorial structure of the CSS-36 presents evidence of being strictly invariant for the groups of men and women in the sequence of proposed invariance models: metric invariance (ΔRMSEA = −0.006), scalar (ΔRMSEA = 0.000), and strict invariance (ΔRMSEA = −0.001).

**Table 6:** Models of invariance according to sex.

Models	*χ* ^2^	df	*p*	SRMR	TLI	CFI	RMSEA	Δ*χ*^2^	Δdf	*p*	ΔRMSEA
Men	2241.18	579	0.000	0.051	0.96	0.96	0.069	-	-	-	-
Women	2603.07	579	0.000	0.044	0.96	0.96	0.067	-	-	-	-
Invariance model											
Configural	2577.48	1,158	0.000	0.040	0.91	0.915	0.042	-	-	-	-
Metric	2236.46	1,188	0.000	0.042	0.93	0.938	0.036	37.43	30	0.165	−0.006
Scalar	2287.66	1,218	0.000	0.042	0.93	0.936	0.036	71.99	30	0.000	0.000
Strict	2312.90	1,254	0.000	0.042	0.94	0.937	0.035	43.14	36	0.193	−0.001

*χ^2^, Chi square; df, degrees of freedom; SRMR, standardized root mean square residual; TLI, Tucker-Lewis index; CFI, comparative fit index; RMSEA, root mean square error of approximation; Δχ^2^, differences in Chi square; Δdf, differences in degrees of freedom; ΔRMSEA, change in root mean square error of approximation; and ΔCFI, change in comparative fix index.*

### Item Response Theory: Gradual Response Model

Two gradual response models (GRM) were adjusted, specifically a 2PLM model for each dimension of the scale based on the two models (Model 1 with six factors and Model 2 with five factors). Table 7 shows the GRM model for each dimension presents acceptable fit indices, while for RMSEAc2 index does not show adequate fit indices in all dimensions. Table 7 shows all the *a* parameters of items of dimensions are above the value of 1, generally considered as good discrimination (Hambleton et al., 2010). Regarding the *b* parameters, in the model with six dimensions, all threshold estimators increased monotonically, as expected. That is, a greater presence of the latent trait is required to answer the higher response categories.

**Table 7:** Discrimination and difficulty parameters for scale items by dimension.

Model	Item	Item parameters					GRM model fit index					
		a	b_1_	b_2_	b_3_	b_4_	C2 (df)	p	RMSEA	SRMSR	TLI	CFI
Danger	E1	1.81	−1.83	−0.58	0.92	2.13	281.77	9	0.148	0.055	0.94	0.96
	E2	3.39	−2.15	−1.15	−0.06	1.01						
	E3	3.57	−2.11	−1.23	−0.34	0.75						
	E4	2.90	−2.02	−1.10	−0.09	1.02						
	E5	1.95	−1.96	−0.64	0.72	1.96						
	E6	2.59	−2.05	−0.91	0.24	1.33						
Socio-economic consequences	E7	3.22	−1.14	−0.24	0.83	1.82	228.67	9	0.132	0.035	0.97	0.98
	E8	2.97	−1.16	−0.30	0.70	1.92						
	E9	3.78	−1.02	−1.19	0.84	1.86						
	E10	3.30	−1.12	−0.29	0.77	1.78						
	E11	3.88	−0.91	−0.19	0.72	1.62						
	E12	2.93	−1.44	−0.41	0.59	1.70						
Xenophobia	E13	2.31	−1.39	−0.56	0.57	1.76	283.84	9	0.148	0.044	0.96	0.97
	E14	3.19	−0.42	0.18	1.03	2.01						
	E15	3.84	−0.90	−0.24	0.73	1.72						
	E16	3.36	−1.11	−0.44	0.64	1.69						
	E17	2.93	−1.13	−0.29	0.69	1.74						
	E18	3.55	−0.48	0.09	0.89	1.82						
Contamination	E19	3.48	−1.22	−0.31	0.62	1.72	221.07	9	0.130	0.031	0.97	0.98
	E20	2.81	−1.69	−0.63	0.37	1.38						
	E21	2.90	−1.35	−0.29	0.71	1.62						
	E22	3.79	−1.31	−0.33	0.67	1.67						
	E23	3.82	−1.09	−0.25	0.74	1.75						
	E24	3.81	−0.82	−0.13	0.94	1.73						
Traumatic stress	E25	3.59	−0.32	0.55	1.58	2.38	100.21	9	0.085	0.024	0.98	0.99
	E26	3.43	−0.14	0.62	1.59	2.41						
	E27	2.51	−0.72	0.21	1.44	2.31						
	E28	3.04	−0.69	0.27	1.33	2.35						
	E29	2.47	−0.25	0.65	1.70	2.56						
	E30	3.14	0.10	0.84	1.78	2.75						
Compulsive checking	E31	2.06	−0.74	0.32	1.45	2.45	232.52	9	0.133	0.061	0.93	0.96
	E32	2.42	−1.16	−0.08	1.16	2.18						
	E33	2.09	−0.95	0.17	1.38	2.36						
	E34	2.46	−0.89	0.13	1.25	2.44						
	E35	1.69	−1.75	−0.37	0.97	2.22						
	E36	1.68	−1.59	−0.24	0.92	2.28						

Model 1 = six items; Model 2 = five items; a = discrimination parameters; and b = difficulty parameters.

**Figure 1:**
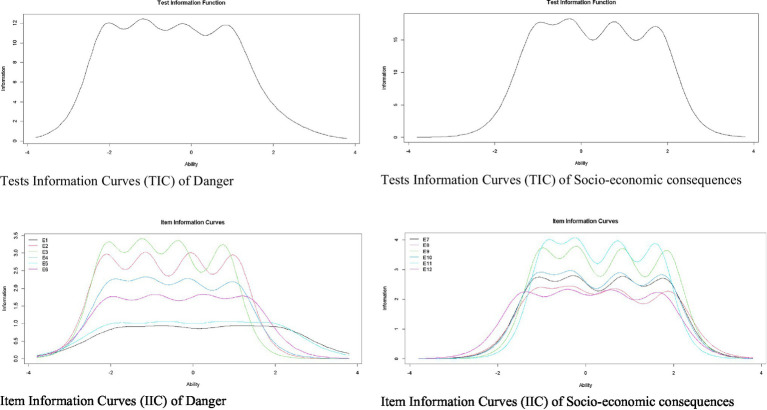
Item and test information curves for danger and socio-economic consequences.

Figure 1 shows Item Information Curves (IIC) and the Test Information Curve (TIC) of the Danger and Socio-economic Consequences dimensions. Regarding the Danger dimension, IIC shows items 2 and 3 are the most accurate for assessing the latent trait. In addition, the TIC shows the factor is most reliable (accurate) in the scale range between −2.5 and 1.5. Regarding the dimension of Socio-economic Consequences, the IIC shows items 11 and 9 are the most accurate in assessing the latent trait; whereas, the TIC shows the factor is more reliable (accurate) in the scale range between −1.5 and 2.5.

**Figure 2:**
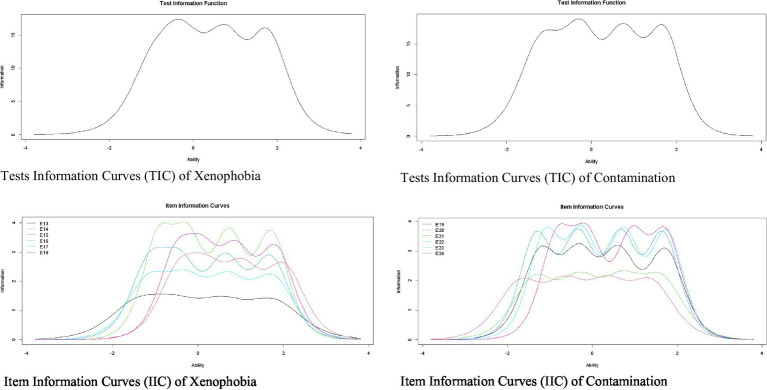
Item and test information curves for the xenophobia and contamination.

Figure 2 shows IIC for the items and TIC for the dimensions of Xenophobia and Contamination. Regarding the Xenophobia dimension, the IIC shows items 15 and 18 are the most accurate in assessing latent trait. In addition, the TIC shows the factor is most reliable (accurate) in the scale range between −1 and 2.5. Regarding the Contamination dimension, the IIC shows items 24, 23, and 22 are the most accurate for evaluating the latent trait. In addition, the TIC shows the factor is most reliable (accurate) in the scale range between −1.5 and 2.5.

**Figure 3:**
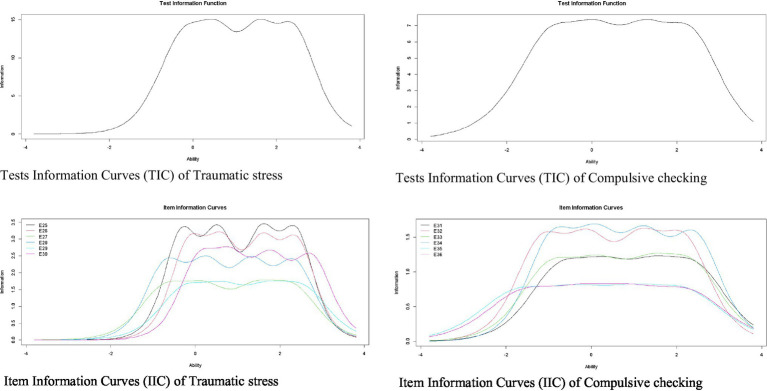
Item and test information curves for traumatic stress and compulsive checking.

Finally, Figure 3 shows the IIC and the TIC for the dimensions of Traumatic Stress and Compulsive Control. Regarding the Traumatic Stress dimension, the IIC shows items 25 and 26 are the most accurate in assessing the latent trait. In addition, the TIC shows the factor is more reliable (accurate) in the scale range between −1 and 3. Regarding the dimension of Compulsive Control, the IIC shows items 34 and 32 are the most accurate to evaluate the latent trait. In addition, the TIC shows the factor is most reliable in the scale range between −1.5 and 3.

## Discussion

During the COVID-19 pandemic, many studies have used instruments, such as the GAD-7, PHQ-9, or DASS-21, which assess mental health indicators in a general way. However, use of these types of measures can generate underestimated or overestimated findings, because they do not aim to identify specific symptoms associated with COVID-19 (Ransing et al., 2020). Seeking to overcome this limitation, instruments have recently been developed to identify mental health symptoms related to COVID-19, such as the CSS-36 (Taylor et al., 2020e). Therefore, the objective of this study is to adapt and evaluate the psychometric evidence of a Spanish version of the CSS-36 in the Peruvian population, using classical psychometric methods, such as CFA, and modern ones, such as the IRT.

With respect to CFA, results indicated the six-dimensional structure of the CSS-36 presents a good fit unlike the five-dimensional model, which presents a lower fit. This result allows us to deduce that, in a Peruvian population, the Danger and Contamination factor are understood as separate factors, unlike the studies in Canada (Taylor et al., 2020e), Persia (Khosravani et al., 2021), and Arabia (Abbady et al., 2021). This is in relation to what Taylor (2019) suggested, when they indicated people use various psychological factors to face the threat of a pandemic, presenting adaptive behaviors, emotions, and defensive reactions that are linked to their psychological vulnerability. On the other hand, it is worth noting that factorial loads are even high, many of them, above what is recommended (Dominguez-Lara, 2018). In this study, factorial loads ranged from 0.69 to 0.92, which is a higher range than reported, for example, in the Canada study, where loads ranged from 0.48 to 0.77. Similarly, reliability of each of the CSS-36 factors is adequate. Values of the alpha coefficient ranged from 0.88 to 0.96, while values of the omega coefficient ranged from 0.87 to 0.96. Findings regarding alpha coefficient values are similar to those reported in Canada (Taylor et al., 2020e), Persia (Khosravani et al., 2021), and Arabia (Abbady et al., 2021). Additionally, in this study, reliability was also reported using the omega coefficient, which is a more appropriate estimation measure because it is based on factorial loads and is not influenced by sample size or number of items on the scale (Ventura-León and Caycho-Rodríguez, 2017).

Once factorial dimensionality of the CSS-36 was established, IRT models were estimated for each factor. Results show that all items in each factor present monotonous values increasing in difficulty parameter. In this sense, a person with low levels of stress associated with COVID-19 will tend to choose a first or second alternative response on the CSS-36; whereas those with greater stress due to COVID-19 will choose a higher response alternative. That is, to respond to higher response options, a greater presence of latent trait (in this case, stress related to COVID-19) will be necessary. Thus, results indicate items reflect the content proposed and any of the response alternatives can be selected while avoid loss of information.

This is an expected finding in instruments that measure psychological distress (Caycho-Rodríguez et al., 2021a). Similarly, item 11 [“I am worried about grocery stores running out of water.” (“Me preocupa que en los almacenes de abarrotes y/o supermercados se acabe el agua.”)] has the best capacity for discrimination. Therefore, this item could more clearly distinguish between individuals with different levels of COVID-19-related stress. Individuals’ responses to item 11 would provide more information about COVID-19 stress, because changes associated with diet have raised concerns about one’s own mood (Laguna et al., 2020). In addition, this is also expected because previous studies indicated fears associated with food supply are greater in the Peruvian population (Gómez-Corona et al., 2021). However, there are population groups which are more likely to experience food insecurity, such as those with low income, people without work or with some type of disability (Loopstra, 2020). In this study, these differences were not examined, so future research should take this into consideration. Based on this, people with COVID-19-related stress will respond more to item 11 compared to those without stress. Regarding fit indices of IRT models, high RMSEA values were observed. However, interpretation of RMSEA for categorical data according to item level is somewhat controversial, since standard values seem to be inadequate to express possible differences generated by number of categories (Maydeu-Olivares and Joe, 2014; Monroe and Cai, 2015).

Previous studies using the CSS-36 and reported differences or similarities in COVID-19 stress between men and women have shown no evidence of MI (Pieh et al., 2020; Xiong et al., 2020; Kolakowsky-Hayner et al., 2021). Results for MI indicate both men and women attribute equal meaning to the stress associated with the COVID-19 pandemic. In addition, CSS-36 items work in the same way, regardless of whether they are answered by men or women. This also suggests predictive relationships between the presence of stress symptoms associated with COVID-19 and other constructs can be significantly compared between the sexes. As mentioned before, absence of evidence for MI in the CSS-6 could lead to errors in the interpretation of comparative results. In this sense, differences in means and associations between groups could be interpreted as the result of methodological problems and not differences associated with the underlying characteristics being assessed.

Despite these results, this study is not free of limitations. First, a sampling was carried out for convenience, not random, which would not allow the results to be generalized to the entire population of Peru. Second, participants were recruited in a relatively short period of time (3 months). This is important to keep in mind, even more so as mental health indicators can vary over time in pandemic situations (Huarcaya-Victoria, 2020). Third, self-report methods were used to obtain results, which may be shaped by social desirability biases. Fourth, an online survey was used, causing the presence of a selection bias, in that only those people who have internet access and experience in carrying out such surveys could access the survey. Despite these limitations, this study has strengths, such as a relatively large sample size (*N* = 1,424 people) and use of classic methods, such as CFA, along with modern ones such as IRT to evaluate psychometric evidence of the CSS-36. This will allow for a greater and better understanding of usefulness of the CSS-36 in the Peruvian context during the COVID-19 pandemic.

In conclusion, the findings indicate that the CSS-36 presents a reliable multidimensional structure with discriminating items that differentiate between those with high and low levels of the latent trait and which is invariant between men and women, which can be used in the Peruvian context and, after future studies, potentially in other similar contexts. In this sense, an appropriate measure in Spanish is provided to measure stress associated with the COVID-19 pandemic. The identification of symptoms of stress during and after the COVID-19 pandemic can provide information to implement intervention programs that allow people to cope with stress during a pandemic and have assertive responses to control measures such as social isolation or strict confinement to which people may be subjected (Duan and Zhu, 2020). The findings suggest the inclusion of the CSS-36 in online mental health assessment systems. This is important in a context of improving mental health services, where technology is a means to deliver mental health services remotely and on a large scale, which is valuable in situations of social distancing. Various professionals use asynchronous interventions for online assessments and recommend mental health applications or online intervention programs (Reay et al., 2020). Even when the COVID-19 pandemic passes, there will likely still be a need for mental health services delivered through technology (Figueroa and Aguilera, 2020). Specifically, internet-based assessment of mental health outcomes would allow for the collection of data to aid in mental health policy formulation before and after the pandemic (Zhou et al., 2020).

## Data Availability Statement

The raw data supporting the conclusions of this article will be made available by the authors, without undue reservation.

## Ethics Statement

Ethical review and approval was not required for the study on human participants in accordance with the local legislation and institutional requirements. The patients/participants provided their written informed consent to participate in this study.

## Author Contributions

MN-G designed and executed the study, analyzed the data, and wrote the paper. AP-A, KG-B, and TC-R collaborated in the study design and paper writing. All authors contributed to the article and approved the submitted version.

## Conflict of Interest

The authors declare that the research was conducted in the absence of any commercial or financial relationships that could be construed as a potential conflict of interest.

## Publisher’s Note

All claims expressed in this article are solely those of the authors and do not necessarily represent those of their affiliated organizations, or those of the publisher, the editors and the reviewers. Any product that may be evaluated in this article, or claim that may be made by its manufacturer, is not guaranteed or endorsed by the publisher.

## References

[ref1] Abbady A. S. El-Gilany A. H. El-Dabee F. A. Elsadek A. M. ElWasify M. Elwasify M. (2021). Psychometric characteristics of the of COVID stress scales-Arabic version (CSS-Arabic) in Egyptian and Saudi university students. Mid. East Curre. Psychiatry 28, 1–9. doi: 10.1186/s43045-021-00095-8

[ref2] Al Maqbali M. Al Sinani M. Al-Lenjawi B. (2021). Prevalence of stress, depression, anxiety and sleep disturbance among nurses during the COVID-19 pandemic: a systematic review and meta-analysis. J. Psychosom. Res. 141:110343. doi: 10.1016/j.jpsychores.2020.110343, PMID: 33360329 PMC7831768

[ref3] Arslan G. Yıldırım M. Tanhan A. Buluş M. Allen K. A. (2020). Coronavirus stress, optimism-pessimism, psychological inflexibility, and psychological health: psychometric properties of the coronavirus stress measure. Int. J. Ment. Heal. Addict. 19, 2423–2439. doi: 10.1007/s11469-020-00337-6, PMID: 32837425 PMC7272108

[ref4] Bakioğlu F. Korkmaz O. Ercan H. (2020). Fear of COVID-19 and positivity: mediating role of intolerance of uncertainty, depression, anxiety, and stress. Int. J. Ment. Heal. Addict. 19, 2369–2382. doi: 10.1007/s11469-020-00331-y, PMID: 32837421 PMC7255700

[ref5] Beaton D. E. Bombardier C. Guillemin F. Ferraz M. B. (2000). Guidelines for the process of cross-cultural adaptation of self-report measures. Spine 25, 3186–3191. doi: 10.1097/00007632-200012150-00014, PMID: 11124735

[ref6] Brown T. A. (2015). Confirmatory Factor Analysis for Applied Research. 2nd *Edn*. New York: Guilford Press.

[ref7] Byrne B. M. (2008). Testing for multigroup equivalence of a measuring instrument: a walk through the process. Psicothema 20, 872–882. 18940097

[ref8] Cai L. Monroe S. (2014). A New Statistic for Evaluating Item Response Theory Models for Ordinal Data (CRESST Report 839). Los Angeles, CA: University of California, National Center for Research on Evaluation, Standards, and Student Testing (CRESST).

[ref9] Caycho T. (2017). Importancia del análisis de invarianza factorial en estudios comparativos en Ciencias de la Salud. Rev. Cub. Educ. Méd. Sup. 31, 1–3.

[ref10] Caycho-Rodríguez T. Valencia P. D. Vilca L. W. Carbajal-León C. Vivanco-Vidal A. Saroli-Araníbar D.. (2021a). Cross-cultural validation of the new version of the coronavirus anxiety scale in twelve Latin American countries. Curr. Psychol. 19, 1–18. doi: 10.1007/s12144-021-02563-0, PMID: 35068911 PMC8765828

[ref11] Caycho-Rodríguez T. Valencia P. D. Vilca L. W. Lee S. A. Carbajal-León C. Vivanco-Vidal A.. (2021b). COVID-19 bereavement in ten Latin American countries: measurement invariance of the pandemic grief scale and its relation to suicidal ideation. Omega doi: 10.1177/00302228211048566, [Epub ahead of print]. PMC10647883 34666552

[ref12] Caycho-Rodríguez T. Vilca L. W. Carbajal-León C. White M. Vivanco-Vidal A. Saroli-Araníbar D.. (2021c). Coronavirus anxiety scale: new psychometric evidence for the Spanish version based on CFA and IRT models in a Peruvian sample. Death Stud. 10, 1–10. doi: 10.1080/07481187.2020.1865480, PMID: 33427098

[ref13] Caycho-Rodríguez T. Vilca L. W. Valencia P. D. Carbajal-León C. Vivanco-Vidal A. Saroli-Araníbar D.. (2021d). Cross-cultural validation of a new version in Spanish of four items of the preventive COVID-19 infection behaviors scale (PCIBS) in twelve Latin American countries. Front. Psychol. 12:763993. doi: 10.3389/fpsyg.2021.763993, PMID: 34867664 PMC8634949

[ref14] Caycho-Rodríguez T. Vilca L. W. Vivanco-Vidal A. Saroli-Araníbar D. Carbajal-León C. Gallegos W. L. A.. (2021e). Assessment of dysfunctional grief due to death from COVID-19 in Peru: adaptation and validation of a Spanish version of the pandemic grief scale. Trends Psychol. 29, 595–616. doi: 10.1007/s43076-021-00091-1 PMC8279032 40477277

[ref15] Chalmers R. P. (2012). Mirt: a multidimensional item response theory package for the R environment. J. Stat. Softw. 48, 1–29. doi: 10.18637/jss.v048.i06

[ref16] Chen F. F. (2007). Sensitivity of goodness of fit indexes to lack of measurement invariance. Struct. Equ. Model. 14, 464–504. doi: 10.1080/10705510701301834

[ref17] Coronavirus Resource Center (2021). COVID-19 Dashboard by the Center for Systems Science and Engineering (CSSE) at Johns Hopkins University (JHU). Available at: https://coronavirus.jhu.edu/map.html (Accessed September 20, 2021).

[ref18] Cronbach L. J. (1951). Coefficient alpha and the internal structure of tests. Psychometrika 16, 297–334. doi: 10.1007/BF02310555

[ref19] Dimitrov D. M. (2010). Testing for factorial invariance in the context of construct validation. Meas. Eval. Couns. Dev. 43, 121–149. doi: 10.1177/0748175610373459

[ref20] Dominguez-Lara S. (2018). Propuesta de puntos de corte para cargas factoriales: una perspectiva de fiabilidad de constructo. Enferm. Clin. 28, 401–402. doi: 10.1016/j.enfcli.2018.06.002, PMID: 30037488

[ref21] Duan L. Zhu G. (2020). Psychological interventions for people affected by the COVID-19 epidemic. Lancet Psychiatry 7, 300–302. doi: 10.1016/S2215-0366(20)30073-0, PMID: 32085840 PMC7128328

[ref22] Figueroa C. A. Aguilera A. (2020). The need for a mental health technology revolution in the COVID-19 pandemic. Front. Psychiatry 11:523. doi: 10.3389/fpsyt.2020.00523, PMID: 32581891 PMC7283500

[ref23] Finney S. J. DiStefano C. (2006). “Non-normal and categorical data in structural equation modeling,” in Structural Equation Modeling. A Second Course. eds. En G. R. Hancock Y. Mueller R. O. (Greenwich, CT: Information Age Publishing), 269–314.

[ref24] Fitzgerald D. A. Wong G. W. (2020). COVID-19: a tale of two pandemics across the Asia Pacific region. Paediatr. Respir. Rev. 35, 75–80. doi: 10.1016/j.prrv.2020.06.018, PMID: 32768308 PMC7319925

[ref25] Gallegos M. Cervigni M. Consoli A. J. Caycho T. Polanco F. Martino P.. (2020). COVID-19 in Latin America: a bibliometric analysis of scientific publications in health. Electron. J. Gen. Med. 17:em261. doi: 10.29333/ejgm/8460

[ref01] Gebhard C. Regitz-Zagrosek V. Neuhauser H. K. Morgan R. Klein S. L. (2020). Impact of sex and gender on COVID-19 outcomes in Europe. Biol. Sex Differ. 11: 29. doi: 10.1186/s13293-020-00304-9, PMID: 32450906 PMC7247289

[ref26] Gómez-Corona C. Rakotosamimanana V. R. Sáenz-Navajas M. P. Rodrigues H. Franco-Luesma E. Saldaña E.. (2021). To fear the unknown: Covid-19 confinement, fear, and food choice. Food Qual. Prefer. 92:104251. doi: 10.1016/j.foodqual.2021.104251, PMID: 34840438 PMC8608550

[ref27] Hambleton R. K. van der Linden W. J. Wells C. S. (2010). “IRT models for the analysis of polytomously scored data: brief and selected history of model building advances,” in Handbook of Polytomous Item Response Models. eds. Nering M. L. Ostini R. (New York, NY: Routledge), 21–42.

[ref28] Hossain M. M. Tasnim S. Sultana A. Faizah F. Mazumder H. Zou L.. (2020). Epidemiology of mental health problems in COVID-19: a review. F1000Research 9:636. doi: 10.35663/amp.2020.371.909, PMID: 33093946 PMC7549174

[ref29] Huarcaya-Victoria J. (2020). Consideraciones sobre la salud mental en la pandemia de COVID-19. Rev. Peru. Med. Exp. Salud Publica 37, 327–334. doi: 10.17843/rpmesp.2020.372.5419, PMID: 32876225

[ref30] Hunsley J. Mash E. J. (2008). A Guide to Assessments That Work. New York: Oxford University Press.

[ref31] Jorgensen T. D. Pornprasertmanit S. Schoemann A. M. Rosseel Y. (2018). semTools: useful tools for structural equation modeling. R package version 0.5-1. Available at: https://cran.r-project.org/web/packages/semTools/semTools.pdf (Accessed September 10, 2021).

[ref32] Khosravani V. Asmundson G. J. Taylor S. Bastan F. S. Ardestani S. M. S. (2021). The Persian COVID stress scales (Persian-CSS) and COVID-19-related stress reactions in patients with obsessive-compulsive and anxiety disorders. J. Obsessive Compul. Relat. Disord. 28:100615. doi: 10.1016/j.jocrd.2020.100615, PMID: 33354499 PMC7746142

[ref33] Kline R. B. (2015). Principles and Practice of Structural Equation Modeling. 4th *Edn*. New York: Guilford Publications.

[ref34] Kolakowsky-Hayner S. A. Goldin Y. Kingsley K. Alzueta E. Arango-Lasprilla J. C. Perrin P. B.. (2021). Psychosocial impacts of the COVID-19 quarantine: a study of gender differences in 59 countries. Medicina 57:789. doi: 10.3390/medicina57080789, PMID: 34440995 PMC8400641

[ref35] Laguna L. Fiszman S. Puerta P. Chaya C. Tárrega A. (2020). The impact of COVID-19 lockdown on food priorities. Results from a preliminary study using social media and an online survey with Spanish consumers. Food Qual. Prefer. 86:104028. doi: 10.1016/j.foodqual.2020.104028, PMID: 32834551 PMC7358165

[ref36] Lee J. H. Lee D. Hyun S. Hong J. S. Kim C. H. Kim W.. (2021). Online mental health assessments of COVID-19 patients in South Korea. Front. Psychol. 12:685445. doi: 10.3389/fpsyt.2021.685445, PMID: 34295275 PMC8290056

[ref37] Leenen I. (2014). Virtudes y limitaciones de la teoría de respuesta al ítem para la evaluación educativa en las ciencias médicas. Invest. Educ. Méd. 3, 40–55. doi: 10.1016/S2007-5057(14)72724-3

[ref38] Loopstra R. (2020). Vulnerability to food insecurity since the COVID-19 lockdown. Available at: https://foodfoundation.org.uk/wp-content/uploads/2020/04/Report_COVID19FoodInsecurity-final.pdf (Accessed September 21, 2021).

[ref39] Lubbe D. Schuster C. (2019). A graded response model framework for questionnaires with uniform response formats. Appl. Psychol. Meas. 43, 290–302. doi: 10.1177/0146621618789394, PMID: 31156281 PMC6512163

[ref02] Maydeu-Olivares A. (2013). Goodness-of-fit assessment of item response theory models. Measurement 11, 71–101. doi: 10.1080/15366367.2013.831680

[ref40] Maydeu-Olivares A. Joe H. (2014). Assessing approximate fit in categorical data analysis. Multivar. Behav. Res. 49, 305–328. doi: 10.1080/00273171.2014.911075, PMID: 26765800

[ref41] McDonald R. P. (1999). Test Theory: A Unified Treatment. New York: Psychology Press.

[ref42] Milfont T. L. Fischer R. (2010). Testing measurement invariance across groups: applications in cross-cultural research. Int. J. Psychol. Res. 3, 111–130. doi: 10.21500/20112084.857

[ref43] Ministerio de Salud (2021). Sala Situacional COVID-19 Perú. Available at: https://covid19.minsa.gob.pe/sala_situacional.asp (Accessed September 15, 2021).

[ref44] Monroe S. Cai L. (2015). Evaluating structural equation models for categorical outcomes: a new test statistic and a practical challenge of interpretation. Multivar. Behav. Res. 50, 569–583. doi: 10.1080/00273171.2015.1032398, PMID: 26717119 PMC4697283

[ref45] Moret-Tatay C. Murphy M. (2022). Anxiety, resilience and local conditions: a cross-cultural investigation in the time of COVID-19. Int. J. Psychol. 57, 161–170. doi: 10.1002/ijop.12822, PMID: 34837393 PMC9011839

[ref46] Muñiz J. (2010). Las teorías de los tests: teoría clásica y teoría de respuesta a los ítems. Papeles Psicól. 31, 57–66.

[ref47] Nochaiwong S. Ruengorn C. Thavorn K. Hutton B. Awiphan R. Phosuya C.. (2021). Global prevalence of mental health issues among the general population during the coronavirus disease-2019 pandemic: a systematic review and meta-analysis. Sci. Rep. 11, 10173–10118. doi: 10.1038/s41598-021-89700-8, PMID: 33986414 PMC8119461

[ref48] Palomino-Oré C. Huarcaya-Victoria J. (2020). Trastornos por estrés debido a la cuarentena durante la pandemia por la COVID-19. Horiz. Med. 20:e1218. doi: 10.24265/horizmed.2020.v20n4.10

[ref49] Passavanti M. Argentieri A. Barbieri D. M. Lou B. Wijayaratna K. Mirhosseini A. S. F.. (2021). The psychological impact of COVID-19 and restrictive measures in the world. J. Affect. Disord. 283, 36–51. doi: 10.1016/j.jad.2021.01.020, PMID: 33516085 PMC7833558

[ref50] Peterson C. H. Peterson N. A. Powell K. G. (2017). Cognitive interviewing for item development: validity evidence based on content and response processes. Meas. Eval. Couns. Dev. 50, 217–223. doi: 10.1080/07481756.2017.1339564

[ref51] Pieh C. Budimir S. Probst T. (2020). The effect of age, gender, income, work, and physical activity on mental health during coronavirus disease (COVID-19) lockdown in Austria. J. Psychosom. Res. 136:110186. doi: 10.1016/j.jpsychores.2020.110186, PMID: 32682159 PMC7832650

[ref04] Prieto-Molinari D. E. Bravo G. L. A. De Pierola I. Victoria-de Bona G. L. Silva L. A. M. Nunez C. S. L.. (2020). Depression and anxiety during the mandatory isolation period due to COVID-19 in Metropolitan Lima. Liberabit 26:e425. doi: 10.24265/liberabit.2020.v26n2.09, PMID: 32470593

[ref52] Putnick D. L. Bornstein M. H. (2016). Measurement invariance conventions and reporting: the state of the art and future directions for psychological research. Dev. Rev. 41, 71–90. doi: 10.1016/j.dr.2016.06.004, PMID: 27942093 PMC5145197

[ref53] R Core Team (2019). A language and environment for statistical computing (R version 3.6.1). R Foundation for Statistical Computing. Available at: https://www.r-project.org/ (Accessed September 10, 2021).

[ref54] Ransing R. Ramalho R. Orsolini L. Adiukwu F. Gonzalez-Diaz J. M. Larnaout A.. (2020). Can COVID-19 related mental health issues be measured? Brain Behav. Immun. 88, 32–34. doi: 10.1016/j.bbi.2020.05.049, PMID: 32470593 PMC7248629

[ref55] Reay R. E. Looi J. C. Keightley P. (2020). Telehealth mental health services during COVID-19: summary of evidence and clinical practice. Australas. Psychiatry 28, 514–516. doi: 10.1177/1039856220943032, PMID: 32722963 PMC7387833

[ref57] Rodriguez-Morales A. J. Gallego V. Escalera-Antezana J. P. Méndez C. A. Zambrano L. I. Franco-Paredes C.. (2020a). COVID-19 in Latin America: the implications of the first confirmed case in Brazil. Travel Med. Infect. Dis. 35:101613. doi: 10.1016/j.tmaid.2020.101613, PMID: 32126292 PMC7129040

[ref58] Rodríguez-Morales A. J. Sánchez-Duque J. A. Hernández Botero S. Pérez-Díaz C. E. Villamil-Gómez W. E. Méndez C. A.. (2020b). Preparación y control de la enfermedad por coronavirus 2019 (COVID-19) en América Latina. Acta Med. Peruana 37, 3–7. doi: 10.35663/amp.2020.371.909

[ref59] Rosseel Y. (2012). lavaan: an R package for structural equation modeling. J. Stat. Softw. 48, 1–36. doi: 10.18637/jss.v048.i02

[ref60] Rothan H. A. Byrareddy S. N. (2020). The epidemiology and pathogenesis of coronavirus disease (COVID-19) outbreak. J. Autoimmun. 109:102433. doi: 10.1016/j.jaut.2020.102433, PMID: 32113704 PMC7127067

[ref61] RStudio Team (2018). RStudio: integrated development environment for R. RStudio, Inc. Disponible en. Available at: https://www.rstudio.com/ (Accessed September 10, 2021).

[ref62] Salari N. Hosseinian-Far A. Jalali R. Vaisi-Raygani A. Rasoulpoor S. Mohammadi M.. (2020). Prevalence of stress, anxiety, depression among the general population during the COVID-19 pandemic: a systematic review and meta-analysis. Glob. Health 16, 1–11. doi: 10.1186/s12992-020-00589-w, PMID: 32631403 PMC7338126

[ref63] Samejima F. (1997). “Graded response model,” in Handbook of Modern Item Response Theory. eds. Van der Linden W. J. Hambleton R. K.. (New York, NY: Springer), 85–100.

[ref64] Schermelleh-Engel K. Moosbrugger H. Müller H. (2003). Evaluating the fit of structural equation models: tests of significance and descriptive goodness-of-fit measures. Methods Psychol. Res. Online 8, 23–74.

[ref65] Scholten H. Quezada-Scholz V. E. Salas G. Barria-Asenjo N. A. Molina R. García J. E.. (2020). Abordaje psicológico del COVID-19: una revisión narrativa de la experiencia latinoamericana. Rev. Int. Psicol. 54:e1287. doi: 10.30849/ripijp.v54i1.1287

[ref66] Schumacker R. E. Lomax R. G. (2015). A Beginner’s Guide to Structural Equation Modeling. 4th Edn. New York, NY: Routledge.

[ref67] Spiteri G. Fielding J. Diercke M. Campese C. Enouf V. Gaymard A.. (2020). First cases of coronavirus disease 2019 (COVID-19) in the WHO European region, 24 January to 21 February, 2020. Euro. Surveil. 25:2000178. doi: 10.2807/1560-7917.ES.2020.25.9.2000178, PMID: 32156327 PMC7068164

[ref68] Squires J. E. Hayduk L. Hutchinson A. M. Cranley L. A. Gierl M. Cummings G. G.. (2013). A protocol for advanced psychometric assessment of surveys. Nurs. Res. Pract. 2013:156782. doi: 10.1155/2013/156782, PMID: 23401759 PMC3562582

[ref69] Stoecklin S. B. Rolland P. Silue Y. Mailles A. Campese C. Simondon A.. (2020). First cases of coronavirus disease 2019 (COVID-19) in France: surveillance, investigations and control measures, January, 2020. Eur. Secur. 25:2000094. doi: 10.2807/1560-7917.ES.2020.25.6.2000094, PMID: 32070465 PMC7029452

[ref03] Taylor S. (2019). Psychology of Pandemics: Preparing for the Next Global Outbreak of Infectious Disease. UK: Cambridge Scholars Publishing.

[ref70] Taylor S. (2021). COVID stress syndrome: clinical and nosological considerations. Curr. Psychiatry Rep. 23, 1–7. doi: 10.1007/s11920-021-01226-y, PMID: 33660068 PMC7927783

[ref71] Taylor S. Landry C. A. Paluszek M. M. Asmundson G. J. (2020d). Reactions to COVID-19: differential predictors of distress, avoidance, and disregard for social distancing. J. Affect. Disord. 277, 94–98. doi: 10.1016/j.janxdis.2020.102289, PMID: 32799109 PMC7413096

[ref72] Taylor S. Landry C. A. Paluszek M. M. Fergus T. A. McKay D. Asmundson G. J. (2020a). COVID stress syndrome: concept, structure, and correlates. Depress. Anxiety 37, 706–714. doi: 10.1002/da.23071, PMID: 32627255 PMC7362150

[ref73] Taylor S. Landry C. A. Paluszek M. M. Fergus T. A. McKay D. Asmundson G. J. (2020e). Development and initial validation of the COVID stress scales. J. Anxiety Disord. 72:102232. doi: 10.1016/j.janxdis.2020.102232, PMID: 32408047 PMC7198206

[ref74] Taylor S. Landry C. A. Rachor G. S. Paluszek M. M. Asmundson G. J. (2020c). Fear and avoidance of healthcare workers: an important, under-recognized form of stigmatization during the COVID-19 pandemic. J. Anxiety Disord. 75:102289. doi: 10.1016/j.janxdis.2020.102289, PMID: 32853884 PMC7434636

[ref75] Taylor S. Paluszek M. M. Rachor G. S. McKay D. Asmundson G. J. (2021). Substance use and abuse, COVID-19-related distress, and disregard for social distancing: a network analysis. Addict. Behav. 114:106754. doi: 10.1016/j.addbeh.2020.106754, PMID: 33310690 PMC8164919

[ref76] Van Agteren J. Bartholomaeus J. Fassnacht D. B. Iasiello M. Ali K. Lo L.. (2020). Using internet-based psychological measurement to capture the deteriorating community mental health profile during COVID-19: observational study. JMIR Ment. Health 7:e20696. doi: 10.2196/20696, PMID: 32490845 PMC7294997

[ref05] Vásquez G. Urtecho-Osorto Ó. R. Agüero-Flores M. Martínez M. J. D. Paguada R. M. Varela M. A.. (2020). Mental health, confinement, and coronavirus concerns: a qualitative study. Interam. J. Psychol. 54:e1333. doi: 10.30849/ripijp.v54i2.1333, PMID: 32627255

[ref77] Ventura-León J. L. Caycho-Rodríguez T. (2017). El coeficiente Omega: un método alternativo para la estimación de la confiabilidad. Rev. Latin. Cien. Soc. 15, 625–627.

[ref78] Viladrich C. Angulo-Brunet A. Doval E. (2017). A journey around alpha and omega to estimate internal consistency reliability. Anal. Psicol. 33, 755–782. doi: 10.6018/analesps.33.3.268401

[ref79] Vindegaard N. Benros M. E. (2020). COVID-19 pandemic and mental health consequences: systematic review of the current evidence. Brain Behav. Immun. 89, 531–542. doi: 10.1016/j.bbi.2020.05.048, PMID: 32485289 PMC7260522

[ref06] Xia Y. Yang Y. (2019). RMSEA, CFI, and TLI in structural equation modeling with ordered categorical data: the story they tell depends on the estimation methods. Behav. Res. Methods 51, 409–428. doi: 10.3758/s13428-018-1055-2, PMID: 29869222

[ref80] Xiong J. Lipsitz O. Nasri F. Lui L. M. Gill H. Phan L.. (2020). Impact of COVID-19 pandemic on mental health in the general population: a systematic review. J. Affect. Disord. 277, 55–64. doi: 10.1016/j.jad.2020.08.001, PMID: 32799105 PMC7413844

[ref81] Yan L. Gan Y. Ding X. Wu J. Duan H. (2021). The relationship between perceived stress and emotional distress during the COVID-19 outbreak: effects of boredom proneness and coping style. J. Anxiety Disord. 77:102328. doi: 10.1016/j.janxdis.2020.102328, PMID: 33160275 PMC7598556

[ref82] Zhou X. Snoswell C. L. Harding L. E. Bambling M. Edirippulige S. Bai X.. (2020). The role of telehealth in reducing the mental health burden from COVID-19. Telemed. e-Health 26, 377–379. doi: 10.1089/tmj.2020.0068, PMID: 32202977

